# Preventing respiratory illness in cerebral palsy: Results of a pilot randomized controlled trial

**DOI:** 10.1371/journal.pone.0325970

**Published:** 2025-06-16

**Authors:** Ryan J. Coller, Kristina Singh-Verdeflor, Jens Eickhoff, Paul J. Chung, Heidi M. Kloster, Christopher C. Cushing, Danielle M. Gerber, Barbara J. Katz, Siem Ia, Teresa Wagner, Roxana Delgado-Martinez, Gemma Warner, Lorena Porras-Javier, Thomas S. Klitzner, Carlos F. Lerner

**Affiliations:** 1 Department of Pediatrics, University of Wisconsin School of Medicine and Public Health, Madison, Wisconsin, United States of America; 2 Department of Biostatistics and Medical Informatics, University of Wisconsin School of Medicine and Public Health, Madison, Wisconsin, United States of America; 3 Department of Health Systems Science, Kaiser Permanente School of Medicine, Pasadena, California, United States of America; 4 Department of Pediatrics and Health Policy & Management, UCLA, Los Angeles, California, United States of America; 5 Clinical Child Psychology Program and Schiefelbusch Life Span Institute, University of Kansas, Lawrence, Kansas, United States of America; 6 Family Voices of Wisconsin, Madison, Wisconsin, United States of America; 7 Department of Pediatrics, David Geffen School of Medicine at UCLA, Los Angeles, California, United States of America; 8 American Family Children’s Hospital, University of Wisconsin Health, Madison, Wisconsin, United States of America; University Medical Centre Ljubljana (UMCL) / Faculty of Medicine, University Ljubljana (FM, UL), SLOVENIA

## Abstract

**Background:**

Respiratory illness is consistently the leading cause of death and hospitalization in severe cerebral palsy (CP). Respiratory Exacerbations-Plan for Action and Care Transitions (RE-PACT) is a just-in-time adaptive intervention to prevent respiratory illness in severe CP. RE-PACT combines early illness detection with rapid clinical response to address varying causes of respiratory illness early enough to modify illness trajectory. This study’s objective was to determine RE-PACT’s feasibility, acceptability, fidelity, and estimated effect size.

**Methods:**

This two-site randomized controlled trial occurred from April 2022-February 2024 in demographically and geographically distinct locations. Caregiver-child pairs were recruited from complex care programs, and children had both gross motor function classification system level 4–5 CP and either pulmonologist care or daily respiratory treatments. Children were randomized to usual care or RE-PACT for six months. Primary outcomes were feasibility, acceptability, and fidelity measures having *a priori* definitions of success. The primary clinical outcome was the severe respiratory illness (SRI) event rate, defined as hospitalizations due to respiratory diagnoses. Clinicaltrials.gov registration is NCT05292365.

**Results:**

Sixty children were enrolled, of which 26 were randomized into RE-PACT. Measures confirmed RE-PACT’s feasibility, acceptability, and fidelity, e.g., text message response rates were 97.5%, and no action planning or clinical responder activities were missed. System usability scale scores were “good to excellent” (mean [SD], 79.5 [11.7]). The RE-PACT SRI event rate (95% confidence interval, CI) was 0.71 (0.36–1.14) per person-year compared to the usual care event rate 1.08 (0.61–1.91) per person-year, a risk ratio of 0.66 (0.28–1.56). Secondary outcomes and qualitative data reinforced RE-PACT’s positive impact.

**Conclusions:**

RE-PACT is a feasible, acceptable intervention that can be delivered with high fidelity to diverse families caring for children with severe CP. These data inform the sample and design characteristics needed for efficacy testing of RE-PACT’s ability to prevent severe respiratory illness.

## Introduction

Severe cerebral palsy (CP), i.e., levels 4–5 in the gross motor function classification system (GMFCS) [1], places children at high risk for serious acute illnesses, such as respiratory, gastrointestinal, and seizure disorders [2]. Respiratory illness is consistently the leading cause of death and hospitalization in severe CP [3,4], accounting for 25% of hospitalizations [2,5] and 59% of deaths [4,6]. Improving these outcomes has been difficult, even though respiratory illness is considered modifiable [7].

Optimal management of respiratory illness in severe CP presents several challenges. Because respiratory illness typically begins at home [8], families have identified the need for crisis management and self-efficacy interventions [9]. Preventing poor outcomes requires, in part, opportunities for families and clinical teams to connect early enough to change trajectory [9–11], which is often difficult for families and clinicians alike. Simultaneously, respiratory illness reflects an assortment of conditions, e.g., pneumonia, pneumonitis, asthma, chronic respiratory failure. Even within a single condition, biologic mechanisms of respiratory illness in severe CP are multifactorial, paralleling other neuromuscular conditions [12–14], including upper airway abnormalities, poor chest wall compliance, inflammatory fibrosis, impaired airway clearance, and respiratory muscle weakness. Moreover, respiratory illness can directly or indirectly result from non-respiratory comorbid and social conditions [7,15], such as seizures, dysphagia, health system navigation barriers, coordination problems, and social determinants of health. These complex realities make static illness plans difficult to develop and implement. For example, an action plan to address wheezing may not help when the child is having a lower respiratory infection, or their parents struggle to reach a provider for guidance.

We hypothesized that a just-in-time adaptive intervention (JITAI) strategy [16] combining early illness detection with customized response could address the proximate causes of respiratory illness in severe CP early enough to modify the illness trajectory and reduce hospitalizations. We designed the Respiratory Exacerbation-Plans for Action and Care Transitions (RE-PACT) intervention [17] to prevent respiratory illness by delivering the right level of support at the right time and in the right manner to meet a child’s current health status and family context. The objective of this study was to conduct a pilot randomized controlled trial (RCT) of RE-PACT to establish its feasibility, acceptability, fidelity, and preliminary effect sizes in two demographically and geographically distinct sites.

## Methods

### Design

This two-site RCT was conducted from April 2022 to February 2024 over three successively larger 6-month “waves”. Between trial waves, interim data were reviewed to aid protocol adjustments to improve implementation. The University of Wisconsin-Madison Institutional Review Board approved this study (2021−1532), written consent was obtained from participants, and the detailed protocol is available in a prior publication [17].

### Setting and sample

Caregivers of children with severe CP were recruited from pediatric complex care program (PCCP) medical homes at the UW Health Kids American Family Children’s Hospital and the UCLA Mattel Children’s Hospital, during three periods (Wave 1: 4/14/2022–7/12/2022; Wave 2: 10/4/2022-11/7/2022; Wave 3: 4/11/2023–8/18/2023). These programs serve similar patients, based on numbers of affected organ systems, subspecialists, and the amount of health services use [18,19].

Study eligibility criteria included being the parent or guardian of a child < 18 years of age with CP GMFCS level 4–5 using a validated caregiver-report instrument [20] and either care from a pulmonologist or daily respiratory treatments. Daily respiratory treatments were defined as oxygen, ventilation, airway clearance devices, or respiratory medications. Caregivers were at least 18 years of age with comfort speaking English or Spanish and a mobile phone capable of sending/receiving text messages. Caregivers received $100 after enrollment and $100 after completion.

We aimed to recruit 10 participants for Wave 1, 20 participants for Wave 2, and up to 60 participants for Wave 3. This sample was not based on sufficient power to establish intervention efficacy but was expected to provide sufficient information to judge feasibility and estimate effect sizes to calculate sample sizes for a subsequent larger RCT. Wave 1 participants were all assigned to the intervention group, not randomized, and were therefore excluded from clinical endpoint assessment. Wave 2 and 3 participants were randomly assigned to either intervention or usual care using 1:1 allocation with random sizes of 2 and 4 and stratified by study site. Randomization was computer generated by a biostatistician with no participant interaction. Random allocation was concealed to research staff conducting recruitment.

### Intervention & procedures

RE-PACT was created by combining and adapting two prior interventions, Plans for Action & Care Transitions (PACT) and Assessing Confidence at Times of Increased Vulnerability (ACTIV) [21,22]. We designed RE-PACT as a prototypical JITAI [16] and integrated substantial family, clinician, and expert engagement throughout [23]. The intervention consists of three core activities: action planning, mobile health (mHealth) monitoring of parent confidence to avoid hospitalization by text message (a JITAI “tailoring variable”, i.e., data from an individual indicating need for intervention) [16], and rapid clinical response during vulnerable periods (S1 Fig) [17].

#### Action planning.

Within 1 month of enrollment, action plans were created with families using the format and process adapted from the original PACT study [21]. The three main elements included (1) a “focus area” for the action plan; (2) “severity levels” corresponding to objective and subjective indicators of baseline (“green”), concerning (“yellow”), and severe (“red”) statuses; and (3) “specific actions” caregivers should take to manage each status. Action plans were offered in English or Spanish, and paper and electronic copies (via the Electronic Health Record’s interactive patient portal) were provided to families in their preferred language.

#### mHealth.

Each participant received weekly text messages asking, “How confident are you that your child can avoid an unplanned hospitalization over the next month?” and responded on a scale from 1–10, with 10 being highest confidence. Text messages were sent at random days and times averaging once weekly (Sunday-Thursday) from 8AM-9PM, with up to 2 reminders sent after 2 hours of non-response. Participants could send responses at any time. These data functioned as a JITAI tailoring variable such that confidence ratings <5 would trigger additional intervention as our prior work indicated that this is the threshold for hospitalization risk [22].

#### Rapid clinical response.

Trained clinical responders, i.e., clinicians such as PCCP physicians, nurses, or advanced practice providers, received email and text notification when participants reported low confidence. Low confidence (ratings <5) or two other tailoring variable triggers—hospital discharge or caregiver phone or electronic message conveying acute respiratory concerns—triggered rapid clinical response within 24 hours to understand the nature of the situation, assist the caregiver in developing solutions, provide clinical guidance, and refer to existing or create new action plans. The responses were documented in the patient’s medical record. Clinical responders followed up with caregivers at least twice over the subsequent two weeks. The same responder worked with the family throughout the study.

#### Usual care.

Each PCCP provides comprehensive interdisciplinary care and care coordination from a team composed of general pediatricians, nurse practitioners, and care coordination personnel (nurses and care coordination assistants) [18,19]. Clinic visits are approximately twice the length of traditional primary care visits, occur at least every 6 months, and include comprehensive care planning, subspecialty co-management, case management, and communication with community services.

### Outcomes

The primary study outcomes were measures of feasibility, acceptability, and fidelity, which we adapted from prior studies [21,22,24–26]. Measures included enrollment rate, time to recruit and deliver the intervention, duration and drop out, intervention use and satisfaction, and the system usability scale [27]. For several measures, we set *a priori* definitions of success (Table 1) which reflected consensus goals set by our research team [17]. At the end of the study, PCCP members were invited to complete a survey to share perceptions about RE-PACT’s efficacy, sustainability, and potential improvements. The primary clinical endpoint was severe respiratory illness (SRI) rate, defined as respiratory diagnosis requiring hospitalization per person-year. Respiratory diagnosis was defined as discharge diagnosis of any of the following: asthma, pneumonia (community or hospital-acquired), bronchiolitis, influenza, upper or lower respiratory tract infection, tracheitis, aspiration pneumonia/pneumonitis, chronic lung disease, or respiratory failure [28]. Secondary clinical outcomes included the numbers of children with at least one severe respiratory illness, at least one hospitalization, systemic steroid courses, systemic antibiotic courses, respiratory ED visits, or death.

**Table 1:** RE-PACT feasibility, acceptability, and fidelity measures.

Feasibility			Success Definition
Recruitment	Days to meet target enrollment size per wave, *median (interquartile range [IQR])*	49.5 (29-118)	<14 days
Intervention onset	Days between intervention start date and first confidence mHealth text assessment, median (IQR)^*^	4 (3.5-7)	<7 days
	Days between randomization and completed action plan, median (IQR)^*^	15.5 (8.5–32)	<30 days
Intervention time	Minutes logged for action planning, mean (standard deviation [SD])^*^	22.8 (10.9)	
	Minutes logged for clinical response, mean (SD)^*^	86.8 (122.2)	
Intervention triggers	Number of intervention triggers per participant, mean (SD)^*^	0.7 (1.0)	
**Acceptability**			
Enrollment	Enrollment rate, patients enrolled/ eligible, approached, *n (%)*	60 (40.3)	>80%
Loss, Drop out	Rate of drop out (active or passive) before six months (# drop out/ # enrolled), *n (%)*	1/60 (1.7)	<10%
Intervention use	Number of months where participants used an action plan, mean (SD)^*^	1.6 (1.6)	
	Number of months where participants interacted with the clinical responder, mean (SD)^*^	1.4 (1.5)	
	Number of participants reporting any use of the action plan, n (%)^*^	23 (64)	
	Number of participants reporting any interaction with the clinical responder, n (%)^*^	22 (61)	
Intervention satisfaction, Participants	Probably or definitely recommends action planning to others, n (%)^*^	25 (71.4)	
	Probably or definitely recommends the texting and clinical response to others, n (%)^*^	27 (77.1)	
	Both action planning and texting/clinical response is moderately important, important, or very important, n (%)^*^	32 (91.4)	
	Probably or definitely want these approaches to continue as a part of regular care, n (%)^*^	23 (65.7)	
	System Usability Scale, mean (SD)^*^	79.5 (11.7)	
	Total number of problems reported with action plans throughout study, sum^*^	1	
	Total number of problems reported with texting or the clinical response throughout study, sum^*^	2	
Intervention satisfaction, Clinical staff^**^	The intervention is quite a bit or extremely likely to prevent hospitalizations for respiratory illness, *n (%)*	8 (100)	
	Probably or definitely want these approaches to continue as a part of regular care, *n (%)*	7 (88)	
	I thought RE-PACT was easy to use, *Agree or strongly agree*, *n (%)*	8 (100)	
	I found the various functions of RE-PACT were well integrated, *Agree or strongly agree*, *n (%)*	7 (88)	
	I would imagine that most people would learn to use RE-PACT very quickly, *Agree or strongly agree*, *n (%)*	7 (88)	
**Fidelity**			
Enrollment duration	Study enrollment duration in months, *mean (SD)*	5.9 (0.5)	6
Action planning	Number of action plans per participant, mean (SD)^*^	1 (0)	≥1
Clinical response visits	Number of clinical response visits completed/ expected by clinical response trigger, (%)^*^		>80%
	Caregiver reported confidence < 5	9/9 (100)	
	Hospital Discharge	9/9 (100)	
	Caregiver calls or messages complex care team	4/4 (100)	
	Multiple triggers	4/4 (100)	
Clinical response follow-ups	Number of clinical response follow-ups completed/ expected by clinical response trigger, (%)^*^		>80%
	Caregiver reported confidence < 5	20/18 (>100)	
	Hospital Discharge	30/18 (>100)	
	Caregiver calls or messages complex care team	9/8 (>100)	
	Multiple triggers	10/8 (>100)	
Cross-over	Number of participants inappropriately receiving intervention, *sum (%)*	0 (0)	0
mHealth texting response rate	Number of text responses/ texts expected, (%)^*^	867/889 (97.5)	>90%
Data collection rates	Data collection completed/ expected by data collection instrument		>95%
	Enrollment surveys, *(%)*	59/ 60 (98.3)	
	Monthly surveys, (%)^*^	210/216 (97.2)	
	Exit surveys, *(%)*	55/ 60 (91.7)	

Definitions of success were set *a priori*. Rows without pre-planned success definitions were intended to be informed by this study’s results.

* These values apply only to participants allocated to the intervention arm.

** Responses from n = 8 pediatric complex care program staff from both study sites (1 nurse, 4 nurse practitioners, 3 physicians)

### Participant characteristics

Participant characteristics included caregiver and child demographics, the child’s number of specialists, presence of selected clinical conditions, and medical devices. Race and ethnicity were included since disparities in access and utilization are known to exist from systemic and structural racism in similar populations [29,30].

### Analysis

Analyses followed the intent-to-treat principle. Feasibility, fidelity, and acceptability measures were summarized with descriptive statistics. A conventional content analysis was conducted for free-text survey responses to identify themes of RE-PACT experiences and influence on caregiving self-efficacy and child health. One research team member reviewed the data, identified codes, and developed themes, which were then refined through iterative team-based discussions.

Although underpowered to assess efficacy for the clinical endpoints, we compared differences between intervention and control group outcomes at six months to estimate effect sizes to inform a future RCT. The SRI (Severe Respiratory Illness) rate per person-year was analyzed with negative binomial (NB) regression modelling to account for overdispersion in the count data. In the primary analysis, univariate NB regression analysis included study arm as a predictor variable and study site to account for the stratified randomization. The observed effect size was quantified by calculating risk ratio (RR) of the SRI rates between the intervention and usual care arms, which were reported along with the corresponding 95% confidence interval. Secondary clinical endpoints were summarized as frequencies and percentages in tabular format and compared between study arms using a chi-square or Fisher’s exact test. Two-sided p-values <0.05 were considered statistically significant. Quantitative analyses were conducted in SAS software (SAS Institute, Cary NC) version 9.4 and qualitative analyses were conducted in Dedoose v9.0.46.

## Results

### Participants

**Fig 1:**
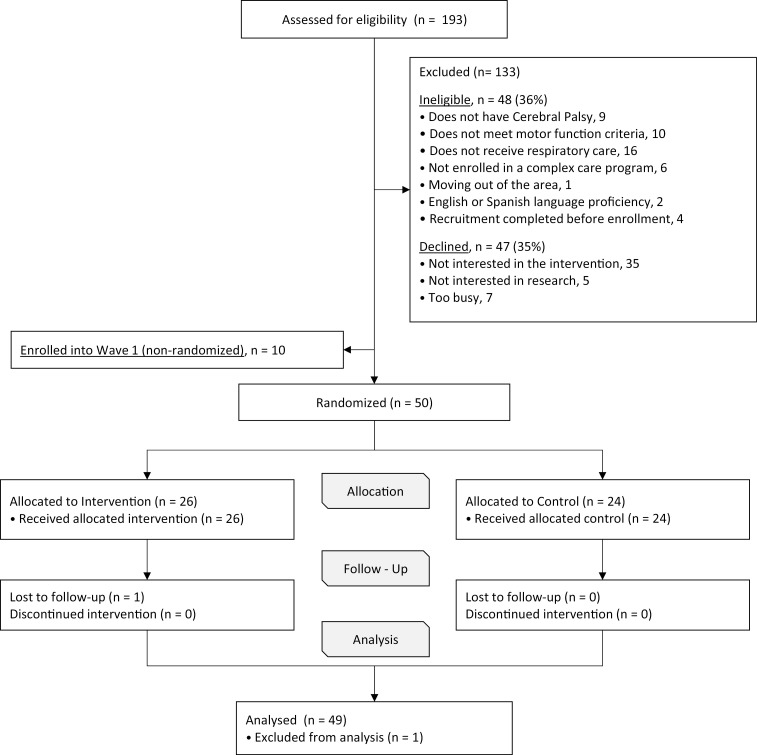
RE-PACT consort diagram.

We enrolled n = 60 caregiver-child dyads, of which n = 26 were randomized to the RE-PACT intervention (Fig 1). Of the randomized participants in waves 2 and 3, caregivers were median (interquartile range, IQR) age 40 (35–46), 32% Hispanic, and 18% participated in Spanish. All income levels and a range of education were represented as were diverse racial/ethnic identities (Table 2). Children were approximately two-thirds male (60%), from all age groups, and had median (IQR) 7 (5–11) subspecialists. Nearly all of the children had (96%) had pulmonologists, and many used vest and/or cough assist devices (58%), oxygen (42%), or non-invasive ventilation (26%). About one-quarter had tracheostomies.

**Table 2:** Demographic and clinical characteristics of caregivers and children in the RE-PACT trial.

	Total n (%)	Intervention^1^ n (%)	Control n (%)
Caregiver characteristics	n = 49^2^	n = 25	n = 24
Caregiver age in years, *median (IQR)*	40 (35-46)	42 (37-48)	37 (34-42)
Race and Ethnicity			
White, Non-Hispanic	24 (49)	12 (48)	12 (50)
Hispanic	15 (31)	8 (32)	7 (29)
Black, Non-Hispanic	4 (8)	1 (4)	3 (13)
Other race, Non-Hispanic	1 (2)	1 (4)	0
Multiracial, Non-Hispanic	2 (4)	2 (8)	0
Not reported	3 (6)	1 (4)	2 (8)
Highest Education			
< 12^th^ grade	5 (10)	3 (12)	2 (8)
GED or some college	24 (49)	10 (40)	14 (58)
Bachelor’s degree	11 (23)	6 (24)	5 (21)
Advanced degree	4 (8)	4 (16)	0
Prefer not to respond or not reported	5 (10)	2 (8)	3 (13)
Income			
< $35,000	15 (31)	6 (24)	9 (38)
$35,000-$49,999	6 (12)	1 (4)	5 (21)
$50,000-$74,999	2 (4)	2 (8)	0
$75,999-$99,999	7 (14)	3 (12)	4 (17)
> $100,000	10 (21)	7 (28)	3 (12)
Not reported	9 (18)	6 (24)	3 (12)
Intervention Language			
English	40 (82)	19 (76)	21 (87)
Spanish	9 (18)	6 (24)	3 (13)
**Child characteristics**	**n = 50**	**n = 26**	**n = 24**
Sex			
Male	30 (60)	19 (73)	11 (46)
Female	18 (36)	7 (27)	11 (46)
Not reported	2 (4)	0	2 (8)
Age			
0–5 y	14 (28)	9 (35)	5 (21)
6–12 y	18 (36)	6 (23)	12 (50)
≥ 13 y	16 (32)	11 (42)	5 (21)
Not reported	2 (4)	0	2 (8)
Primary Insurance Type^3^			
Public insurances	39 (78)	21 (81)	18 (75)
Private insurances	7 (14)	4 (15)	3 (12)
Other insurances	2 (4)	1 (4)	1 (4)
Not reported	2 (4)	0	2 (8)
Number of subspecialists, *median (IQR)*	7 (5-11)	7 (5-11)	8 (5-9)
Number of children experiencing the following diseases:			
Seizures	32 (64)	18 (69)	14 (58)
Asthma	14 (28)	7 (27)	7 (29)
Chronic lung disease	19 (38)	8 (31)	11 (46)
Number of children using the following services or technology:			
Pulmonologist	48 (96)	25 (96)	23 (96)
Gastrostomy tube	40 (80)	20 (77)	20 (83)
Gastrostomy-Jejunostomy tube and/or Nissen fundoplication	22 (44)	13 (50)	9 (38)
Home Oxygen	21 (42)	10 (38)	11 (46)
Vest and/or Cough Assist	29 (58)	14 (54)	15 (63)
CPAP and/or BiPAP	13 (26)	7 (27)	6 (25)
Tracheostomy with or without ventilator	11 (22)	5 (19)	6 (25)

^1^ ‘Intervention’ in this table includes all participants from waves 2 and 3.

^2^ Caregivers were allowed to enroll multiple children in the study.

^3^ The ‘Public Insurances’ category includes participants with both public and private insurance. The ‘Other Insurances’ category includes participants who selected “Does not have health insurance”, “Don’t Know”, or “Prefer not to answer.”

CPAP = continuous positive airway pressure; BiPAP = bilevel positive airway pressure

### Feasibility, acceptability, and fidelity

Most measures of feasibility, acceptability, and fidelity met our team’s success definitions (Table 1). The primary challenge was achieving the goal enrollment, due to a larger rate (36%) of ineligibility from respiratory or GMFCS criteria. About 40% of those approached and eligible were enrolled, over median (IQR) 49.5 (29–118) recruitment days. About one-third declined to participate.

Action planning and mHealth text messaging began within the first two study weeks. Text message response rates were 97.5%. Twenty-six total rapid clinical response triggers occurred, and no clinical response activities were missed. On average, participants had one intervention trigger during their enrollment. Action planning and clinical responder activities required mean (SD) 22.8 (10.9) and 86.8 (122.2) minutes per enrollee, respectively. Survey data elements were very complete (96.4%) across all instruments. There were no detected treatment crossovers, adverse events, or data infrastructure problems. The single patient who did not complete the study lost eligibility due to being discharged from their PCCP. Participants and clinical staff reported high levels of satisfaction with RE-PACT as well as interest in seeing the intervention continue in routine care (Table 1). Mean (SD) system usability scale scores were 79.5 (11.7), which is in the “good” to “excellent” range by convention; and comparable to scores for an iPhone or Amazon [31]. Protocol changes made between waves were summarized (S1 Text).

### Clinical outcomes

With respect to the primary clinical outcome, children in the RE-PACT intervention group had a SRI rate (95% CI) of 0.71 (0.36–1.41) per person-year compared to a rate of 1.08 (0.61–1.91) per person-year in the control group. The risk ratio (95% CI) was 0.66 (0.28–1.56) for intervention versus control, p = 0.35 (Table 3). The secondary clinical outcomes (Table 4) all favored intervention; but as anticipated, they were not statistically significant. No intervention participants had ED visits for respiratory illness during the study.

**Table 3:** Primary clinical endpoint in the RE-PACT trial.

	Severe Respiratory Illness Rate (per person-year)^1^		Risk Ratio^1^		
	Estimate	95% CI	Estimate	95% CI	p-value
**Intervention, n = 25**	0.71	0.36-1.41	0.66	0.28-1.56	0.35
**Control, n = 24**	1.08	0.61-1.91			

^1^Based on a zero-inflated negative binomial model with study site as stratification factor

**Table 4:** Secondary clinical endpoints in the RE-PACT trial.

	Intervention n = 25	Control n = 24	Risk Ratio	p-value
	n	n	(95% CI)	
**Severe respiratory illness,** children with at least one, n (%)	8 (32)	11 (46)	0.70 (0.34-1.43)	0.32
**Hospitalization,** children with at least one, n (%)	2 (8)	6 (25)	0.32 (0.07-1.43)	0.13
**Systemic Steroids Treatment**, children with at least one, n (%)	3 (12)	3 (12)	0.96 (0.21-4.30)	0.99
**Systemic Antibiotic Treatment**, children with at least one, n (%)	5 (20)	6 (25)	0.80 (0.28-2.28)	0.68
**Respiratory ED Visit**, children with at least one, n (%)	0 (0)	3 (12)	NA	0.11
**Death**, n (%)	0 (0)	0 (0)	NA	0.99

### Qualitative results

Using participant and clinical personnel data, we identified three themes describing the intervention’s influence on caregiving self-efficacy and child health: RE-PACT (1) functions as tool to build skill, confidence, and adaptability; (2) promotes the maintenance of stable child health; and (3) facilitates collaboration and connection to the clinical team (Table 5). For example, caregivers reported greater confidence and decision-making skills from RE-PACT. Action planning supported confidence in crisis management, and clinical responders empowered caregivers to adapt and resolve health crises. Several families mentioned that action plans improved symptom recognition and earlier intervention, which “gets the situation under control faster”, and “has helped keep [the child] home during illnesses.” Actions plans oriented all providers and caregivers to be “on the same page working towards a final goal.” Many caregivers valued the consistent connection to their clinical team via text, stating that “it is quick, easy and efficient.” Consistent with RE-PACT’s intention to tailor intervention activities to families’ caregiving experience and needs, a few caregivers noted that they may not have to use these tools often but that it was “nice to know it was available if necessary.”

**Table 5:** Themes, subthemes, and illustrative quotes on RE-PACT’s feasibility, acceptability, fidelity, and effect on caregiving self-efficacy and child health from enrolled caregivers and clinical personnel involved in the study.

Theme	Subtheme	Illustrative Quote
Functions as a tool to build skill, confidence, and adaptability	Action plans were widely regarded as vital tools in crisis management, providing clear signals and steps to follow and increasing caregiver knowledge.	“Plan for the worst, hope for the best. Having the plan and equipment ready to go makes it so much better when it’s needed. Emotions and cognition will clash during a medical episode and this will take the guess-work out of it- just review and follow the plan.”
	Clinical responders empowered caregivers to resolve health crises, adapting clinical guidance to the family’s current circumstance.	“Es más fácil cuando nuestros hijos están enfermos y nosotros como padres preocupados nos da ventaja al comunicarnos con los especialistas para reflexionar y tomar la decisión de asistir a la sala de emergencias” *(“It’s easier when our kids are sick and we, as worried parents, are given the advantage of communicating with the specialists to think it over and make the decision to go to the emergency department.”)*
	The intentional use of action planning and clinical response facilitated identification of caregiver knowledge gaps that may have otherwise been missed.	“The conversations with families during action planning and JITAI visits are extremely helpful in determining caregiver learning needs/gaps. This sometimes gets overlooked during busy clinic visits.” – RE-PACT Clinical Provider
	Caregivers with high confidence/ activation may not need to utilize these tools as often as others.	“With having myChart, I didn’t feel it was necessary to have the text messages.”
Promotes the maintenance of stable child health	Action plans improved recognition of disease symptoms, allowing intervention at home early enough to prevent emergency department or hospital visits.	“Gracias por el plan de salud de mi hijo a ayudado bastante para mantener más estable su salud y evitar asistir a la sala de emergencia.” *(“Thanks for the action plan for my child. It’s helped so much to keep his health more stable and avoid going to the ED”)*
Facilitates the collaboration and connection to clinical Team	Action plans organized multiple parties (family, school, clinicians, etc.) to the same set of actions and goals to optimize the child’s health.	“It is super helpful to have something visual on hand for parents, caregivers, and schools.”
	The texting aspect provided a sense of security for families, knowing they have a quick connection to the clinical team if needed.	“Porque brinda seguridad de que qué hay un monitoreo al paciente.” *(“Patient monitoring provides a sense of security”)*
	Caregivers appreciated that clinical responders proactively reached out to families in the weeks following a JITAI visit.	“My son contracted Covid for the first time and we were followed up on multiple occasions throughout the week to ensure he was doing well.”

## Discussion

In this two-site pilot RCT, the RE-PACT intervention was feasible, acceptable, and delivered with high fidelity among participants having diverse language, income, and education experiences. By combining proactive action planning, simple mHealth monitoring, and rapid clinical response, RE-PACT aimed to reduce severe respiratory illness events in children with GFMCS level 4–5 CP. Although the study was not powered to evaluate efficacy, the study data preliminarily support the hypothesis that RE-PACT may reduce these events, and the results will be used to estimate sample sizes needed for a large efficacy trial.

Novel intervention approaches such as RE-PACT are urgently needed for severe CP, since respiratory illness remains the leading cause of death and hospitalization despite being considered modifiable [3,4,7,32]. We described our theoretical model and RE-PACT’s hypothesized causal pathway to reduce respiratory illness events in an earlier study [23]. While we were underpowered to confirm the validity of the relationships of the causal pathway in this pilot RCT, doing so remains an important goal of a larger follow-up trial. We are not aware of other interventions designed to simultaneously address prevention, surveillance, and acute management of respiratory illness in CP. In fact, a 2019 systematic review of interventions to manage respiratory disease in young people with CP concluded that only weak or absent evidence exists for most interventions [33]. Moreover, the authors did not identify a single intervention to prevent respiratory disease.

A recent expert consensus statement on preventing and managing respiratory illness in young people with CP advocated for four specific activities: early identification of risk factors; regular assessment of risk; effective partnerships between multidisciplinary teams, families, and individuals with cerebral palsy; and proactive treatment of respiratory disease [15]. RE-PACT’s components reflect these key activities. Moreover, by promoting clinical, communication, and social supports for caregivers in vulnerable moments, the adaptive and holistic approaches to action planning and rapid clinical response could plausibly lead to broader health improvements in severe CP as well, such as quality of life, mortality, and caregiving stress or benefit [8,34]. JITAI designs should efficiently address the family’s needs and provide the right amount or type of support at the right time [16]. Simultaneously, the JITAI approach may efficiently use clinician effort by calibrating intervention doses to the times and intensities indicated by the child’s current circumstances. This hypothesis will be tested in future implementation studies.

Our encouraging feasibility, acceptability, and fidelity results were enriched by qualitative data as well as the perspectives of both participants and clinicians. These observations were consistent with our prior studies of PACT [21] and ACTIV [22,35], two interventions that were integrated into RE-PACT’s design. Among children with medical complexity, which includes children with severe CP, we have observed that digital interventions designed to support family-delivered care at home are received well by families as indicated by measures of feasibility, acceptability, fidelity, and usability [24–26]. Although JITAI designs are still relatively uncommon in pediatrics, other researchers have also found this design to be promising among individuals with serious chronic illness and caregivers. For example, a pilot RCT of an mHealth JITAI to improve self-management for caregivers of individuals with spinal cord injury, Huntington’s disease, or hematopoietic cell transplant observed similarly high feasibility, acceptability, and fidelity despite frequent data collection and text messaging [36].

Amidst our positive results, opportunities for ongoing improvement remain. Although we originally intended to recruit up to 90 individuals, the total number of eligible children at the two clinical sites ended up smaller than initially estimated. Despite this reality, the study was positively received, and this experience will greatly help plan for realistic recruitment into a large-scale trial. A minority of participating families reported not needing RE-PACT in routine care. We suspect this reflects a few different scenarios. In some cases, the child’s respiratory status may be generally stable, and six months may not provide sufficient time to experience the intervention in a manner that reinforced its value, e.g., they may not have had any illnesses or low confidence periods. In other cases, family mastery of respiratory illness monitoring and management may already be high, and communication channels with providers may already meet their needs. Interventions such as RE-PACT may best serve families earlier in their journeys as caregivers. Similarly, RE-PACT activities might best serve children who are cared for by multiple caregivers beyond a primary parent/guardian. Finally, it is possible that family and interventionist interactions did not yield action plans or clinical responses that added sufficient value to them. Some components of the intervention might have been too complex (suggested in a handful of individual responses to specific items in the system usability scale). These concepts will be important to explore in subsequent research. Continuing to adapt RE-PACT to be maximally usable and for the children and families who may benefit the most will be a central future direction. For example, if *post-hoc* analyses suggest that families speaking Spanish had greater difficulty or less satisfaction with the intervention, RE-PACT should be adapted to better meet this population’s needs.

This study has several limitations. As expected, we were underpowered to determine RE-PACT’s efficacy. Allocation concealment was not possible due to the nature of the intervention activities. Because randomization occurred at the level of the family, rather than the clinic, it is difficult to know whether clinician behavior for usual care patients differed during the study. However, action planning and text messaging were easy to limit to intervention participants, and such a situation would bias results towards the null. In future trials we plan to randomize at the clinic level using cluster randomized or stepped-wedge designs to further minimize this threat. Generalizability is limited to the population of families recruited from two complex care programs, and implementation in primary care would likely have greater staffing complexity. In the future, we hope to expand RE-PACT to populations of children with CP beyond complex care programs and from even greater geographically, linguistically, and culturally diverse settings. We also intend to extend the duration beyond 6 months to understand seasonal variation and longer-term benefits in addition to including a broader outcome set, such as quality of life, milder respiratory illnesses, and caregiver health. The scalability and sustainability of RE-PACT remains unknown and will be the subject of future implementation trials. Intervention costs primarily relate to staff time for action planning and clinical responses; however, not all RE-PACT work is new since already spend time contingency planning and triaging family concerns. Rigorous economic evaluations of RE-PACT will be important.

Despite these limitations, our two-site pilot RCT suggests that RE-PACT is feasible and acceptable and can be delivered with high fidelity. While the clinical endpoint was not expected to reach statistical significance in this study, the results were in the expected direction and magnitude of effect. These preliminary data will support the rigorous design of a large-scale efficacy or effectiveness-implementation hybrid trial.

## Supporting information

S1 Fig RE-PACT intervention activities. (PDF)

S1 Text RE-PACT protocol refinements. (DOCX)

S1 Checklist CONSORT checklist. (DOCX)

## Abbreviations

JITAI: Just-in-Time Adaptive Intervention
CP: Cerebral Palsy
GMFCS: Gross Motor Function Classification System
RE-PACT: Respiratory Exacerbation-Plans for Action and Care Transitions
