# Supplementary material for: Preventing respiratory illness in cerebral palsy: Results of a pilot randomized controlled trial
Source: PLoS One. 2025 Jun 16;20(6):e0325970. doi: 10.1371/journal.pone.0325970 (PMC12169566; doi:10.1371/journal.pone.0325970)
Supplement: S1 Fig — (PDF) [file pone.0325970.s001.pdf]

Respiratory Exacerbation - Plans for Action & Care Transitions (RE-PACT): Intervention Activities

Action Planning

At study entry, clinical providers create an action plan based on the child's known contributors to respiratory illness

The plan is reviewed with families and providers incorporate feedback

Physical and electronic copies are provided in the family's preferred language

Child Name:<br>Clinician Author:<br>Pulmonary Agreement:	Revision Date:<br>Creation Date:<br>Update Due:
Plan Focus:	Care Team Phone Number
**Green Zone**	
Signs	*Actions*
**Yellow Zone**	
Breathing is harder, faster	Give breathing medication<br>Increase suction
**Red Zone**	
Not tolerating nutrition	Call complex care
If you see these, call 911 or go to the emergency room:	
Critical Signs	

Goal is to increase caregiver knowledge, confidence, and resources

mHealth Surveillance

Every week, caregivers receive the above text message prompt to their personal cell phone

Caregivers receive a tailored response based on their reported confidence level

"How confident are you that your child can avoid an unplanned hospitalization over the next month? Please respond of a scale from 1 ("not confident") to 10 ("fully confident"). If your child is currently hospitalized, please repond by texting "h". Thank you!"

5

Review your action plans! As always, if things get worse, reach out to your child's healthcare providers directly

Goal is to 1) provide a recurring and accesible link for the caregiver to the care team and 2) easily signal to clinicians that a child with Cerebral Palsy is at high risk of respiratory illness and hospitalization

Just In Time Adaptive Intervention Clinical Response

Clinical responders will reach out to the family within 24 hours after any of the three identified prompts have occurred

The clinical responder identifies the nature of the situation and lets the caregiver develop solutions, while assisting with clinical guidance and potential obstacles as needed

Text response < 5

Caregiver messages or calls care team

Hospital Discharge

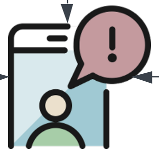

The clinical responder references established action plans and may create new ones as appropriate

The clinical responder will follow up with the caregiver at least two times within the subsequent weeks

Goal is to increase caregiver knowledge, confidence, and skills in collaboration with the care team
