# Supplementary material for: Preventing respiratory illness in cerebral palsy: Results of a pilot randomized controlled trial
Source: PLoS One. 2025 Jun 16;20(6):e0325970. doi: 10.1371/journal.pone.0325970 (PMC12169566; doi:10.1371/journal.pone.0325970)
Supplement: S1 Text — (DOCX) [file pone.0325970.s002.docx]

Supplement

RE-PACT Protocol Refinements

Between trials waves, several improvements were implemented in the RE-PACT intervention. The boundaries of the rapid clinical response activities were further defined in wave two to more clearly define when a response event was considered complete. The original workflow consisted of a triage visit and two follow-ups over the course of two weeks, with additional visits as needed. However, because patients may experience multiple or prolonged medical issues that extend beyond the expected scope of the clinical response, we modified the protocol to better define issues falling within or beyond the RE-PACT scope, including when additional issues arise during rapid clinical response events. This allowed our clinical responders to better determine when to end or extend rapid clinical response events.

Additionally, in wave two, our texting platform was refined to optimize participant data entry and functionality. Participants can send confidence texts at any time, even unprompted; and any reported confidence level < 5 initiates a rapid clinical response visit. Because the original platform was built to record only one confidence level per week, this modification allowed recording of additional values that could be submitted by participants spontaneously. In addition, we implemented a standard response to inactive participants and unknown phone numbers, stating “Messages are not currently being monitored. Please notify your health provider if you have any health concerns. Call 911 if this is a medical emergency.” These adjustments allowed study staff and providers to better respond to participants’ needs, while ensuring that no inactive participants used the texting platform inadvertently or expected a response to an unmonitored text message they submitted.
